# QUESTIONS RELATED TO SCREENING OF EMOTIONAL/BEHAVIORAL PROBLEMS IN PRESCHOOLERS

**DOI:** 10.1590/1984-0462/;2018;36;1;00018

**Published:** 2018

**Authors:** Marina Monzani da Rocha

**Affiliations:** a Programa de Pós-graduação em Distúrbios do Desenvolvimento, Universidade Presbiteriana Mackenzie, São Paulo, SP, Brasil.

The assessment of behavioral problems among preschoolers was considered controversial for a long time due to the fear of turning difficulties inherent to development into pathologies. However, the strategy of “wait and see” did not work for most of the cases^1^ and, currently, several studies show that the problems from the first childhood period may remain throughout life when without any intervention.^2^

The study of Santos and Celeri^3^ about screening mental health problems in preschool children, which was published in the current edition of *Revista Paulista de Pediatria*, was developed with the aim of favoring an early assessment in the context of essential health care. The authors reinforce the importance of cultural adaptation of instruments that have been developed in other countries to facilitate the early identification of problems,^4^ which results in better prognosis.

The Strengths and Difficulties Questionnaire (SDQ)^5^ used by the authors^3^ is internationally recognized as a fast and effective instrument for screening the difficulties presented by children aged between 2 and 4. Emotional symptoms, conduct problems, hyperactivity/inattention, peer-relationship problems and prosocial behaviors are assessed based on the 25 items answered in a Likert scale. Results found by Santos and Celeri^3^ confirmed that the SDQ allows the identification of cases presenting problems within a “normal”, “borderline” or “abnormal” range in Brazil. In addition, they indicate significant and positive correlations between the scales of SDQ and preschool Child Behavior Checklist (CBCL/1.5-5),^6^ which is another internationally known instrument for the assessment of small children’s behavioral problems.

The SDQ may be useful to screen cases requiring a complete assessment to verify the presence of mental health problems and follow-up for intervention. However, it is noteworthy that the adherence of the health basic care team is a question to be overcome so the use of this kind of instrument is effective to screen problems and consequently to promote population’s health.
